# High-flow nasal oxygen vs. standard oxygen therapy for patients undergoing transcatheter aortic valve replacement with conscious sedation: a randomised controlled trial

**DOI:** 10.1186/s13741-023-00300-8

**Published:** 2023-04-14

**Authors:** S. Scheuermann, A. Tan, P. Govender, M. Mckie, J. Pack, G. Martinez, F. Falter, S. George, A. A. Klein

**Affiliations:** 1 Department of Anaesthesia and Intensive Care, Royal Papworth Hospital, Cambridge, UK; 2 MRC Biostatistics Unit, University of Cambridge, Cambridge, UK

**Keywords:** Sedation, Oxygen, Cardiac surgery, Cardiology, High-flow nasal oxygen, Peri-operative care

## Abstract

**Background:**

Minimally invasive surgery is becoming more common and transfemoral transcatheter aortic valve replacement is offered to older patients with multiple comorbidities. Sternotomy is not required but patients must lie flat and still for up to 2–3 h. This procedure is increasingly being performed under conscious sedation with supplementary oxygen, but hypoxia and agitation are commonly observed.

**Methods:**

In this randomised controlled trial, we hypothesised that high-flow nasal oxygen would provide superior oxygenation as compared with our standard practice, 2 l min^−1^ oxygen by dry nasal specs. This was administered using the Optiflow THRIVE Nasal High Flow delivery system (Fisher and Paykel, Auckland, New Zealand) at a flow rate of 50 l min^−1^ and FiO_2_ 0.3. The primary endpoint was the change in arterial partial pressure of oxygen (pO_2_) during the procedure. Secondary outcomes included the incidence of oxygen desaturation, airway interventions, the number of times the patient reached for the oxygen delivery device, incidence of cerebral desaturation, peri-operative oxygen therapy duration, hospital length of stay and patient satisfaction scores.

**Results:**

A total of 72 patients were recruited. There was no difference in change in pO_2_ from baseline using high-flow compared with standard oxygen therapy: median [IQR] increase from 12.10 (10.05–15.22 [7.2–29.8]) to 13.69 (10.85–18.38 [8.5–32.3]) kPa vs. decrease from 15.45 (12.17–19.33 [9.2–22.8]) to 14.20 (11.80–19.40 [9.7–35.1]) kPa, respectively. The percentage change in pO2 after 30 min was also not significantly different between the two groups (*p* = 0.171). There was a lower incidence of oxygen desaturation in the high-flow group (*p* = 0.027). Patients in the high-flow group assigned a significantly higher comfort score to their treatment (*p* ≤ 0.001).

**Conclusion:**

This study has demonstrated that high flow, compared with standard oxygen therapy, does not improve arterial oxygenation over the course of the procedure. There are suggestions that it may improve the secondary outcomes studied.

**Trial registration:**

International Standard Randomised Controlled Trial Number (ISRCTN) 13,804,861. Registered on 15 April 2019. https://doi.org/10.1186/ISRCTN13804861

## Background

Transfemoral transcatheter aortic valve replacement (TAVR) has become an established therapy for high-risk patients with severe aortic stenosis (Rosengart et al. 2008). General anaesthesia was the preferred anaesthetic technique in the early days of TAVR, but it has largely been replaced by local anaesthesia and conscious sedation (Miles et al. 2016). Patients undergoing TAVR often have multiple cardiorespiratory risk factors that put them at an increased risk of hypoxia with complications, such as confusion or an exacerbation of pulmonary hypertension (Balanika et al. 2014), especially as they are required to lie flat and still for an extended period of time (Mayr et al. 2015). Lying flat may be difficult for patients with impaired cardiac function as cardiac failure causes pulmonary edema and dyspnea. Also, patients with respiratory disease or patients who are obese may not be able to lie flat and may become hypoxic. In addition, the deployment of the new aortic valve may require rapid cardiac pacing and standstill with no cardiac output which may impair oxygen delivery to the body and make the patient restless (Mayr et al. 2016).

Various sedation techniques have been described, with most supplying oxygen via either nasal cannulae or face mask (Mayr et al. 2015). High-flow nasal oxygen (HFNT) allows the delivery of heated and humidified oxygen at an inspiratory fraction of oxygen (FiO_2_) of between 0.21 to 1.0, in a flow rate of up to 60 l min^−1^ (Drake 2018). Emerging evidence shows that HFNT is effective in various clinical settings such as in the treatment of acute heart failure (Kang et al. 2019; Carratalá Perales et al. 2011; Roca et al. 2013), after cardiac surgery (Corley et al. 2011; Parke et al. 2013) and during procedural sedation (Ben-Menachem et al. 2020; Lucangelo et al. 2012; Lin et al. 2019; Mazzeffi et al. 2020; Schumann et al. 2016; Yi et al. 2020). Besides providing a higher degree of patient comfort by supplying warmed and humidified gas to maintain muco-ciliary functions (Nishimura 2015), HFNT increases pharyngeal pressure by 3 cmH_2_0 and generates an effect similar to continuous airway positive pressure (CPAP) (Nishimura 2016).

We designed this randomised controlled study to evaluate clinical outcomes using HFNT in patients undergoing transfemoral TAVR under conscious sedation. The primary hypothesis was that HFNT would improve oxygenation in this group of patients, as measured by arterial partial pressure of oxygen (pO_2_) during the procedure. Secondary outcomes included the number of episodes of desaturation, the type of airway interventions by the anaesthetist, comfort levels of patients as determined by movement during the procedure, cerebral oxygen saturations, peri-operative oxygen therapy duration, hospital length of stay and patient satisfaction scores.

## Methods

**Fig. 1:**
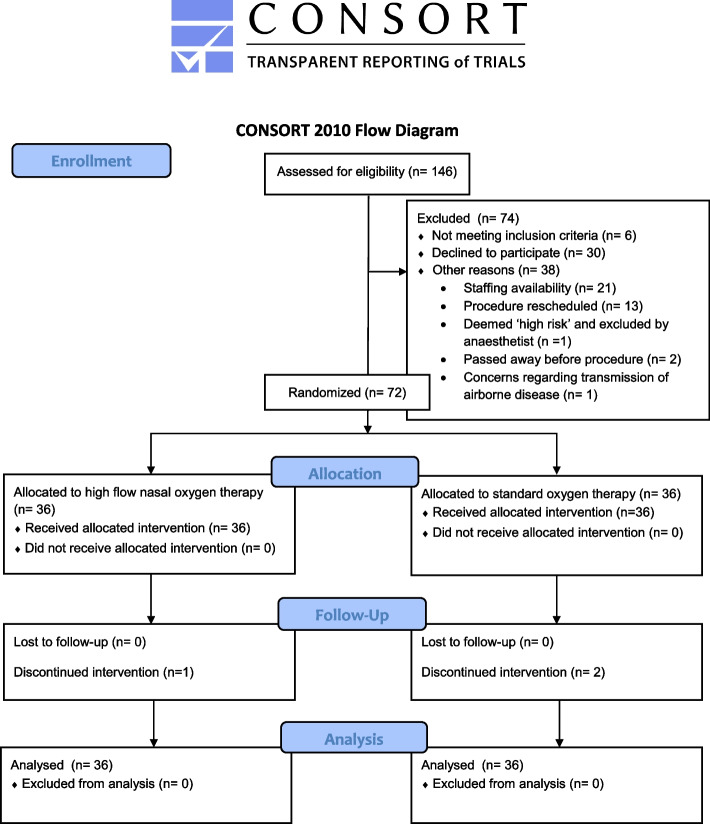
Study flow diagram

The study protocol was approved by the East of England Cambridge East Ethics Committee and all patients provided written informed consent. This was a single-centre, prospective randomised controlled trial conducted at the Royal Papworth Hospital, Cambridge, UK, a specialist cardiothoracic hospital. Patients with nasal septal defects, those who were participating in another randomised controlled trial and those who were unable to speak English or had special communication needs were not included. Patients undergoing elective TAVR under sedation were randomly allocated in a 1:1 ratio using Sealed Envelope™ (https://www.sealedenvelope.com/) online randomisation (unrestricted) to standard oxygen therapy (SOT) or HFNT. Allocation of treatment arm was done by an investigator before the commencement of the procedure. The transcatheter aortic valve procedure was performed in the cardiac catheter laboratory using a transfemoral approach and percutaneous access via the femoral artery with retrograde valve placement (Fig. 1).

Patients, physicians and nurses caring for patients were not blinded to the study interventions as blinding would not have been possible due to the nature of the intervention. The researcher collecting data including blood gas measurements (primary outcome) and interviewing patients was blinded.

Physiological monitoring according to the Association of Anaesthetists standards was applied to all patients (Klein et al. 2021). Cerebral oximetry was measured using the Masimo O3 regional oximetry (Masimo, Irvine, CA, USA). A peripheral intravenous cannula was sited and an ultrasound-guided ilio-inguinal and fascia iliaca block was performed on the side of the procedure using 2 mg kg^−1^ of levobupivacaine by the anaesthetist.

Conscious sedation/analgesia is defined as “a drug-induced depression of consciousness during which patients respond purposefully to verbal commands, either alone or accompanied by light tactile stimulation” (Miles et al. 2016). In our conscious sedation protocol, a low-dose remifentanil infusion was commenced at 0.05mcg kg^−1^ min^−1^ and titrated to achieve a Ramsey sedation scale of 2 to 3 (Ramsay et al. 1974). No other premedication or sedative was given to patients. Oxygen therapy was started after commencement of sedation therapy.

Participants in the HFNT group were given warm and humidified oxygen using the Optiflow THRIVE Nasal High Flow delivery system (Fisher and Paykel, Auckland, New Zealand). Oxygen was administered starting at an FiO_2_ of 0.3 and flow rate of 50 l min^−1^. Continuous capnography monitoring was also used throughout the procedure according to Association of Anaesthetists standards using an attachment to the nasal cannulae provided by the manufacturer.

Participants in the SOT group received 2 l min^−1^ oxygen therapy via standard nasal cannulae (not heated or humidified) and capnography and FiO_2_ was measured using the gas in-line monitoring system of the anaesthetic machine. The FiO_2_ using this protocol was 0.3 (the same as the HFNT group). At any stage during the study, the anaesthetist could manage the patient’s airway in any way they deemed to be appropriate in the best interest of the patient. This included changing to an alternative oxygen delivery strategy.

The cardiologist secured further access with a standard arterial sheath in the femoral artery and a venous sheath in the femoral vein. A 14–16-Fr sheath for valve insertion was placed in the contralateral femoral artery. All procedures utilised Edwards SAPIEN 3 (Edwards Lifesciences, Irvine, CA, USA) or Medtronic CoreValve Evolut PRO (Medtronic, Minneapolis, MN, USA) heart valve system. Transvenous rapid ventricular pacing using a balloon-tipped pacing catheter inserted via the femoral vein was used to facilitate rapid pacing for valve deployment. Transthoracic echocardiography was used to assess valve function before and after the intervention.

The primary outcome was the change in arterial oxygenation, as measured by pO_2_ between the first and second arterial blood gas measurements from the femoral arterial sheath. The first arterial blood gas was obtained as soon as the cardiologist obtained access in the femoral artery and the second was obtained 30 min later. This was the only arterial access in these patients. Both measurements were taken with the patient receiving HFNT or SOT as per group allocation and at the same FiO_2_. Secondary outcomes include the number of episodes of desaturation, as defined as an SpO_2_ of < 93% for > 10 s or a decrease of > 5% from the baseline for > 10 s; the type of airway interventions by the anaesthetist; the number of times the patient reached for the oxygen delivery device; cerebral desaturation during valve deployment, as defined as regional SO_2_ < 50% or decrease in baseline > 20% (Slater et al. 2009); peri-operative oxygen therapy duration; hospital length of stay; and patient satisfaction scores. Blood gas analysis, oxygen device settings, vital signs, cerebral oximetry and sedation score of patients were collected at 30-min intervals after arterial access was obtained.

After the procedure, patients were transferred to the post-anaesthesia recovery unit (PACU) until ready for discharge to the ward. The criteria used for patient discharge was a post-anaesthetic discharge scoring system (PADS) score of > 9. Patients were interviewed within 24 h after the procedure and asked to rate the comfort level of the oxygen delivery device.

We reviewed data in TAVR patients who received sedation with remifentanil from an audit from 2018 and the mean (SD) arterial pO_2_ was 10.0 (5.2) kPa using SOT. We hypothesised that HFNT would lead to a clinically significant improvement of 35% during the procedure. Following these assumptions, a sample size of 34 patients in each arm would be sufficient to detect a difference between study groups at 80% power and a significance level of 0.05. Assuming a dropout rate of 5%, the final sample size was set at 36 patients per group. Data analysis was performed using an intention-to-treat approach. Categorical variables were compared using Fisher’s exact test. Normality was checked using Shapiro–Wilk test and by assessment of skewness and its standard error. Comparison between groups were analysed using Student’s *t-*test where assumptions were met, and Wilcoxon rank sum test if the data was not normally distributed. Repeated measurements were analysed with Wilcoxon signed rank test. All tests were two-tailed and statistical significance was accepted at *p* < 0.05. Statistical analysis was performed using R version 4.0.1 (R Foundation for Statistical Computing, Vienna, Austria).

## Results

A total of 72 patients who underwent transfemoral TAVR between June 2019 and October 2020 were enrolled into the study. None of the participants crossed over to the other treatment arm. Three patients did not complete the procedure: the valve was not deployed in two patients (HFNT group) and one patient passed away as a result of a complication of the procedure (HFNT group). Three patients required conversion to general anaesthesia: one patient in the SOT group developed unstable supraventricular tachycardia and required synchronised DC cardioversion; another in the SOT group had difficulties with valve deployment and required multiple rapid pacing episodes; and one patient in the HFNT group required transesophageal echocardiography. The dose of remifentanil used for sedation during the procedure was similar in both groups, median (IQR [range]) in HFNT group 0.30 (0.24–0.42 [0.14–0.85]) mg vs. SOT group 0.37 (0.26–0.46 [0.10–0.66]) mg, *p* = 0.454. Procedure time was also similar in both groups, HFNT 117.00 (102.50–131.00 [74.0–151.0]) min vs. SOT 115.50 (101.00–135.50 [73.0–218.0]) min, *p* = 0.954 (Table 3).

The two groups had similar baseline characteristics (Table 1) except that twice as many patients had undergone previous cardiac surgery in the HFNT group compared with the SOT group (15% vs. 7%, respectively). None of the patients required continuous positive airway pressure therapy and one patient in the HFNT group required home oxygen therapy.

**Table 1:** Baseline characteristics in patients randomly allocated to high-flow nasal oxygen therapy (HFNT) or standard oxygen therapy (SOT) during sedation for transcatheter aortic valve replacement. Values are mean (SD), number (proportion) or median (IQR [range])

Variable	High-flow nasal oxygen therapy *n* = 36	Standard oxygen therapy *n* = 36
Age; years	83.19 (5.73)	81.80 (6.65)
Sex		
Male	23 (63.9%)	21 (58.3%)
Female	13 (36.1%)	15 (41.7%)
Body mass index; kg m^−2^	26.10 (3.93)	29.01 (5.90)
Body surface area; m^2^	1.82 (0.20)	1.87 (0.24)
Euro-SCORE	3.06 (1.88–4.89 [0.72–30.82])	3.08 (1.98–4.64[1.01–31.35])
ASA physical status		
2	4 (11.1%)	3 (8.3%)
3	18 (50.0%)	19 (52.8%)
4	14 (38.9%)	14 (38.9%)
Smoking status		
Current smoker	2 (5.6%)	2 (5.6%)
Never smoker	22 (61.1%)	18 (50.0%)
Previous smoker	12 (33.4%)	16 (44.4%)
Asthma	3 (8.3%)	3 (8.3%)
COPD	3 (9%)	3 (9%)
Inhaler therapy	6 (16.7%)	4 (11.1%)
Previous cardiac surgery	15 (41.7%)	7 (19.4%)
Oxygen saturation on room air, %	96.61 (2.35)	96.86 (2.45)
Baseline NIRS, L, %	63.28 (5.59)	63.14 (8.79)
Baseline NIRS, R, %	63.11 (7.07)	63.72 (7.10)

*SD* standard deviation, *IQR* interquartile range, *ASA* American Society of Anesthesiologists, *COPD* chronic obstructive pulmonary disease, *NIRS* near infra-red spectroscopy

**Fig. 2:**
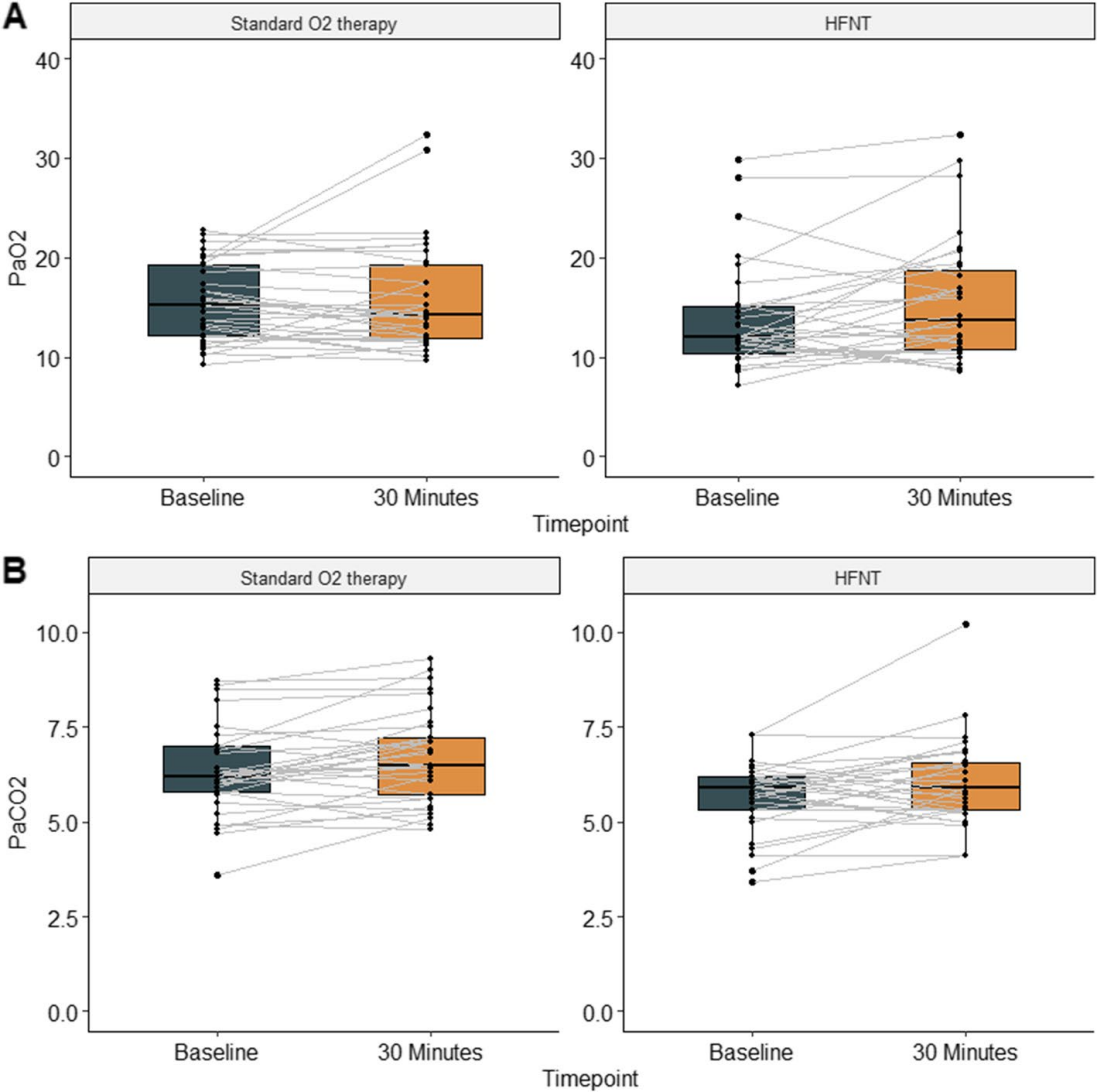
Change in arterial partial pressure of oxygen (pO_2_) and arterial partial pressure of carbon dioxide (pCO_2_) from 0 to 30 min during transfemoral transcatheter aortic valve implantation (TAVR) in patients randomly allocated to high-flow nasal oxygen therapy (HFNT) or standard oxygen therapy (SOT)

**Fig. 3:**
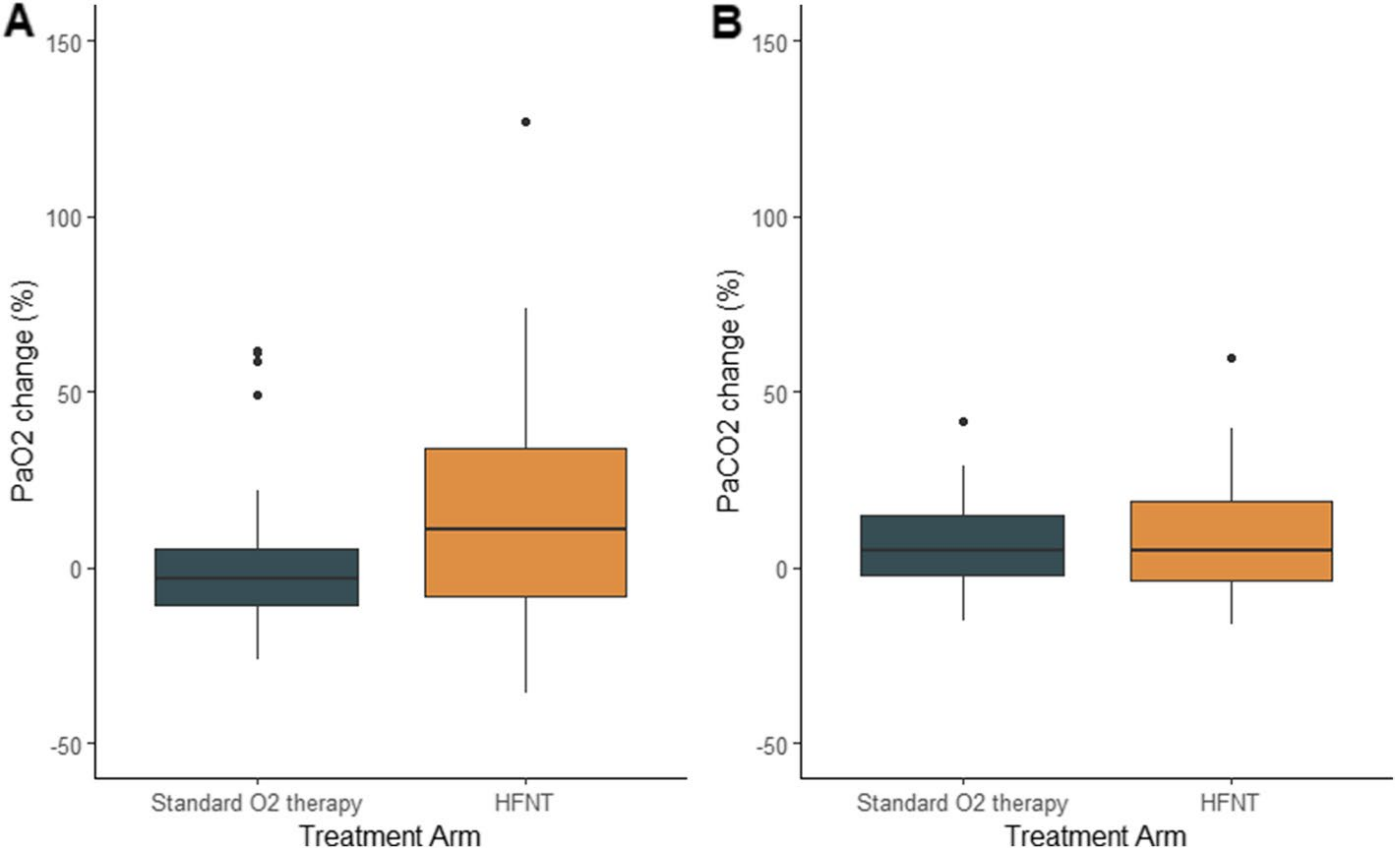
Percentage change in arterial partial pressure of oxygen (pO_2_) and arterial partial pressure of carbon dioxide (pCO_2_) in both high-flow nasal oxygen (HFNT) and standard oxygen therapy (SOT) groups from 0 to 30 min during transfemoral transcatheter aortic valve implantation (TAVR) in patients randomly allocated to HFNT or SOT

Table 2 and Figs. 2 and 3 illustrate the blood gas analysis results. All patients had their first arterial gas analysis after insertion of the femoral arterial line. This was mean (SD) 51 (16) minutes after the pre-surgery checklist had been completed. pO_2_ at baseline was lower in the HFNT compared with the SOT group, median [IQR (range)] 12.10 (10.05–15.22 (7.2–29.8)) vs. 15.45 (12.17–19.33 (9.2–22.8)) kPa. The FiO_2_ was 0.3 in both groups and there was no difference in the percentage change in pO_2_ after 30 min between the two groups (*p* = 0.171) (Table 3).

**Table 2:** Intra-operative blood gas analysis in patients randomly allocated to high-flow nasal oxygen therapy (HFNT) or standard oxygen therapy (SOT) during sedation for transcatheter aortic valve replacement. Values are median (IQR [range])

Variable	Oxygen therapy	Baseline	30 min	*p* value
pO_2;_ kPa	Standard oxygen therapy	15.45 (12.17–19.33 [9.2–22.8])	14.20 (11.80–19.40 [9.7–35.1])	0.69
	High-flow nasal oxygen therapy	12.10 (10.05–15.22 [7.2–29.8])	13.69 (10.85–18.38 [8.5–32.3])	0.067
pCO_2;_ kPa	Standard oxygen therapy	6.15 (5.65–6.93 [2.0–8.7])	6.0 (5.65–7.30 [4.8–9.3])	0.0044
	High-flow nasal oxygen therapy	5.85 (5.18–6.23 [3.4–7.3])	5.85 (5.30–6.53 [4.1–10.2])	0.043
FiO_2_	Standard oxygen therapy	0.3 (0.3–0.3 [0.28–0.5])	0.3 (0.3–0.3 [0.29–1.0])	0.388
	High-flow nasal oxygen therapy	0.3 (0.3–0.3 [0.3–0.4])	0.3 (0.3–0.4 [0.3–0.5])	0.296

*SD* Standard deviation, *IQR* Interquartile range, *pCO*_*2*_ Arterial partial pressure of carbon dioxide, *pO*_*2*_ Arterial partial pressure of oxygen, *FiO*_*2*_ Inspiratory fraction of oxygen

**Table 3:** Secondary outcomes in patients randomly allocated to high-flow nasal oxygen therapy (HFNT) or standard oxygen therapy (SOT) during sedation for transcatheter aortic valve replacement. Values are number (proportion) or median (IQR [range])

Variable	High-flow nasal oxygen therapy *n* = 36	Standard oxygen therapy *n* = 36	*p* value
pO_2_ percentage change from baseline to 30 min	9.62 (− 8.04, 30.57 [− 36.09 to 127.27])	− 2.73 (− 10.82, 5.71[− 26.51 to 97.19])	0.171
pCO_2_ percentage change from baseline to 30 min	4.84 (− 3.71, 17.86 [− 16.13 to 59.46])	5.00 (− 2.74, 14.95 [− 15.52 to 51.02])	0.802
Number of desaturations			0.027
0	31 (86.1%)	24 (66.7%)	
1	4 (11.1%)	3 (8.3%)	
> 1	1 (2.8%)	9 (25.0%)	
Cerebral desaturation*			0.221
Yes	4 (11.8%)	9 (25.0%)	
No	30 (88.2%)	27 (75.0%)	
Number of times patient reaches for oxygen delivery device			0.003
0	34 (94.4%)	23 (63.9%)	
≥ 1	2 (5.6%)	13 (36.1%)	
Comfort level			< 0.001
Excellent	17 (48.3%)	3 (8.6%)	
Good	13 (37.1%)	10 (28.6%)	
Fair	5 (14.3%)	19 (54.3%)	
Poor	0	3 (8.6%)	
Throat dryness			0.484
Severe	1 (2.9%)	1 (2.9%)	
Moderate	10 (28.6%)	15 (42.9%)	
Slight	7 (20.0%)	8 (22.9%)	
Not at all	17 (48.6%)	11 (31.4%)	
Abdominal bloating			0.360
Severe	0	0	
Moderate	0	2 (5.7%)	
Slight	3 (8.6%)	1 (2.9%)	
Not at all	32 (91.4%)	32 (91.4%)	
PACU oxygen therapy			0.123
Yes	14 (40.0%)	22 (61.1%)	
Discharge from PACU with oxygen therapy			0.778
Yes	7 (20.0%)	9 (25.0%)	
PACU stay; min	54.00 (36.25–77.0 [8.0–210.0])	52.50 (39.50–86.25 [15.0–252.0])	0.694
Duration of oxygen therapy; min	119.00 (102.00–170.00 [0–2237.0])	151.00 (111.00–271.00 [78.0–7091.0])	0.209
Hospital stay; days	3.09 (2.24–6.29 [1.33–18.2])	3.55 (2.20–5.49 [1.05–18.4])	0.936
Duration of procedure; min	117.00 (102.50–131.00 [74.0–151.0])	115.50 (101.00–135.50 [73.0–218.0])	0.954

*IQR* Interquartile range, *pCO*_*2*_ Arterial partial pressure of carbon dioxide, *pO*_*2*_ Arterial partial pressure of oxygen, *PACU* Post anaesthesia recovery unit

^*^Valve was not deployed in two patients in the HFNT group

Other study secondary outcomes are shown in Table 3. There was a statistically significantly reduced incidence of oxygen desaturation in the HFNT group (*p* = 0.027). Furthermore, five patients in the SOT group required escalation of oxygen delivery device from nasal cannula to a face mask, versus none in the HFNT group. Four patients in the HFNT group demonstrated cerebral desaturation during valve deployment (11.8%) compared with nine in the SOT group (25.0%), *p* = 0.221. Furthermore, three patients in the SOT group experienced syncope compared with none in the HFNT group. Two patients in the standard group were observed to be restless and agitated, as compared with one in the HFNT group.

Patients described HFNT as being more comfortable, with fewer patients reaching for the oxygen delivery device (*p* = 0.003) and giving it a significantly higher comfort score when interviewed after the procedure (*p* ≤ 0.001). There was no significant difference between the two groups in oxygen requirements in PACU (*p* = 0.123) or duration of hospital stay (*p* = 0.936). There were no postoperative complications related to the use of either oxygen delivery device. No patient died in either group during the first 30 days after TAVR.

## Discussion

We have shown no difference in change in arterial oxygenation over the course of the TAVR procedure in patients who received HFNT or SOT while sedated. Regarding secondary outcomes, fewer patients who received HFNT desaturated. Patients who received HFNT reported feeling more comfortable.

The treatment for aortic stenosis is aortic valve replacement, which has traditionally required sternotomy and cardiopulmonary bypass. Minimally invasive TAVR without sternotomy has become the standard of care for patients with aortic stenosis at increased surgical risk (Baumgartner et al. 2018). Nevertheless, TAVR remains a high-risk procedure when applied to patients who are poor surgical candidates. Around 3250 TAVR cases were performed across centres in the UK in the year 2016 (Ludman 2019) and this is increasing yearly. Transfemoral TAVR is increasingly being done while patients receive conscious sedation (Mayr et al. 2015), with observational study data showing benefits such as an improvement in hemodynamic stability and shorter hospital and ICU stay (Ehret et al. 2017). Hence, it is important to review our anaesthetic practice.

Hypoxia is likely to be more common in patients undergoing transfemoral TAVR due to both patient- and procedure-related factors. Patients are often elderly and have multiple comorbidities (Trauzeddel et al. 2021). Up to a third of them have chronic lung disease (Howard et al. 2019). Furthermore, the procedure takes up to 2 h and patients are required to lie still in the supine position on a reasonably hard and narrow table. The reduction in functional residual capacity further predisposes them to hypoxia (Coonan and Hope 1983), which is often worsened by the need for procedural sedation. As a result of oxygen desaturation, patients regularly become restless and agitated. Therefore, we decided to study whether HFNT would increase arterial pO_2_ and reduce the incidence of intra-operative adverse events.

One of the mechanisms by which HFNT is proposed to improve oxygenation is by alveolar recruitment (Drake 2018). This results from the generation of positive airway pressure, although the magnitude of this effect is dependent on the patient having their mouth closed (Chanques et al. 2013). We did not notice the expected improvements in blood gases despite high oxygen flows. This may be because patients did not have their mouths closed or this is not seen in sedated patients for another reason. In procedures such as bronchoscopy (Ben-Menachem et al. 2020; Lucangelo et al. 2012), gastroscopy (Lin et al. 2019; Mazzeffi et al. 2020) and endoscopic retrograde cholangiopancreatography (Schumann et al. 2016) in which an improvement in oxygenation with the use of HFNT has been shown, the presence of an endoscope may have produced the same effect as having the mouth closed.

High-flow nasal therapy did not have a significant effect on carbon dioxide partial pressure in our study, with percentage change in pCO_2_ levels increasing similarly over time in both the HFNT and SOT groups. Achieving a sufficiently high flow rate is critical to reduce the anatomic dead space in order to maximise CO_2_ clearance (Möller et al. 2017). Douglas et al. noted that increasing the HFNT flow rate from 40 to 60 l min^−1^ brought the pCO_2_ down to within the normal range (Douglas et al. 2018), suggesting that a higher O_2_ flow rate than that used in our study (50 l min^−1^) may have a greater effect on CO_2_ clearance.

In contrast to the lower rates of desaturation in our HFNT patient group, this was not proven in morbidly obese patients undergoing colonoscopy (Riccio et al. 2019). However, there is increasing evidence that the high concentration oxygen therapy used in this study may be detrimental by causing hypoventilation and, hence, hypercapnia (Pilcher et al. 2017). This suggests that hypoventilation may be the cause of hypoxia in morbidly obese patients receiving HFNT. In contrast, our patient cohort has a much lower BMI of 27.6 (5.2) kg m^−2^ (mean (SD)), suggesting that HFNT may be less useful in morbidly obese patients.

Patient comfort level and satisfaction score in many of these studies produced mixed results (Roca et al. 2010; Maggiore et al. 2014), with many suggesting no added benefit of HFNT (Ben-Menachem et al. 2020; Lucangelo et al. 2012; Yilmazel Ucar et al. 2020). This is in contrast to our study which suggests that HFNT has an added advantage of improving the comfort level in patients. This could have been due to the humidification effect of HFNT as seen by the lower incidence of throat dryness in the HFNT group.

HFNT has been shown to have beneficial effects in cardiac patients both in the setting of the treatment of acute heart failure (Kang et al. 2019; Carratalá Perales et al. 2011; Roca et al. 2013) and in the treatment of postoperative hypoxemia after cardiac surgery. In a recent case report, HFNT was used to treat desaturation in a patient undergoing percutaneous balloon aortic valvuloplasty under sedation (Sakazaki et al. 2019). In addition to respiratory benefits, HFNT also resulted in hemodynamic improvements in patients with heart failure (Roca et al. 2013). This is a potential added benefit for patients undergoing transfemoral TAVR, many of whom have heart failure due to severe aortic stenosis.

### Limitations

We have shown no difference in arterial oxygenation in patients who received HFNT or SOT during sedation for TAVR. This may have been because we did not recruit enough patients to see a true difference as the difference was smaller than that used in our power calculation (35%). The study was not powered for the secondary outcomes so these are exploratory and must be interpreted with caution and future studies should look to confirm their findings. Also, our groups differed with respect to baseline pO_2_; we are not sure of the reason for this but it is like to be a random finding. The pO_2_ tended to decrease slightly 30 min later in the SOT group and increase in the HFNT but this was not statistically significant when comparing the two groups—this could also be due to type-2 error.

Secondly, arterial blood gases were only able to pick up pO_2_ and pCO_2_ values at certain time-points and we only managed to capture data at two time-points in most patients. The first arterial gas analysis was obtained at a mean (SD) of 51 (16) minutes after the patient had been checked in, allowing for only one more arterial gas analysis before the end of the procedure.

Also, this study investigated HFNT using a single delivery device at a FiO_2_ of 0.3 and flow rate of 50 l min^−1^. Differences might be seen with alternative HFNT delivery devices or different FiO_2_ and flow rates, as shown in studies demonstrating the beneficial effects of a higher flow rate on CO_2_ clearance (Mauri et al. 2017).

## Conclusions

We have shown that HFNT, compared with SOT, did not improve arterial oxygenation in patients undergoing transfemoral TAVR under sedation over the course of the procedure. There are suggestions that it may improve the secondary outcomes studied, which are hypothesis-generating for future studies.

## Data Availability

The datasets used and/or analysed during the current study are available from the corresponding author on reasonable request.

## Abbreviations

CPAP: Continuous airway positive pressure
FiO_2_: Inspiratory fraction of oxygen
HFNT: High-flow nasal oxygen
PACU: Post-anaesthesia recovery unit
PADS: Post-anaesthetic discharge scoring system
pCO_2_: Arterial partial pressure of carbon dioxide
pO_2_: Arterial partial pressure of oxygen
SOT: Standard oxygen therapy
TAVR: Transfemoral transcatheter aortic valve replacement
